# Liposomes as Carriers for the Delivery of Efavirenz in Combination with Glutathione—An Approach to Combat Opportunistic Infections

**DOI:** 10.3390/app12031468

**Published:** 2022-01-29

**Authors:** Vanaja Kenchappa, Ruoqiong Cao, Vishwanath Venketaraman, Guru V. Betageri

**Affiliations:** 1 College of Pharmacy, Western University of Health Science, Pomona, CA 91766, USA; 2 College of Osteopathic Medicine of the Pacific, Western University of Health Science, Pomona, CA 91766, USA

**Keywords:** liposomes, efavirenz, glutathione, HIV infection, THP-1 cells, oxidative stress

## Abstract

Human immunodeficiency virus (HIV)-infected individuals display an enhanced production of reactive oxygen species (ROS). This reduction of antioxidant capacity in host tissues has been related to the decrease in total levels of ROS scavengers such as glutathione (GSH). Prevention of opportunistic infections due to a weakened immune system is becoming a key strategy along with HIV elimination. Research in these directions is clearly warranted, especially a combination of antiretrovirals and antioxidants to ameliorate oxidative stress, improve intracellular uptake and target viral reservoirs. Hence, we aimed to formulate liposomes loaded with the antiretroviral drug efavirenz (EFA) in the presence of glutathione, as these carriers can be engineered to enhance the ability to reach the target reservoirs. The goal of the present work was to investigate the intracellular uptake of EFA-loaded liposome (with and without GSH) by human monocytic leukemia cells (THP-1 cells) and examine cell viability and ROS scavenging activity. Results obtained provided significant data as follows: (i) treatment with EFA and GSH combination could enhance the uptake and reduce cytotoxicity; (ii) encapsulation of EFA into liposomes increased its levels in the macrophages, which was further enhanced in the presence of GSH; (iii) delivery of EFA in the presence of GSH quenched the intracellular ROS, which was significantly higher when delivered via liposomes. Data revealed that a combination of EFA and GSH encompasses advantages; hence, GSH supplementation could be a safe and cost-effective treatment to slow the development of HIV infection and produce an immune-enhancing effect.

## 1. Introduction

According to the World Health Organization, at the end of 2020, about 37.7 million (30.2–45.1 million) people were infected with the human immunodeficiency virus (HIV); 680,000 [480,000–1.0 million] people died from HIV-related causes and 1.5 million [1.0–2.0 million] people have acquired HIV [1]. HIV mainly targets immune cell types such as CD4+ T cells, monocytes, macrophages, and natural killer (NK) cells [2,3]. Innumerable opportunistic infections associated with HIV infections include Kaposi sarcoma, Pneumocystic pneumonia, Herpes virus and *Mycobacterium tuberculosis* (*M. tb*), all of which could result in poor patient survival [3]. These infections flourish due to a compromised immune system, which follows the depletion of CD4+ T cells.

In recent years, mycobacterial infections have increased due to growing numbers of highly susceptible immunocompromised individuals arising from the AIDS epidemic. Oxidative stress and immunological dysfunction are believed to adversely impact mycobacterial outcomes, resulting in higher risks of disease relapse and mortality and a greater propensity for developing drug-resistant infections [4–7]. Mycobacterial co-infection is a leading cause of mortality in HIV patients and a major contributor to antimicrobial resistance. Efavirenz (EFA) is a drug of choice to treat HIV patients with mycobacterial infections [8]. EFA is an antiretroviral, non-nucleoside reverse transcriptase inhibitor (NNRTI) used specifically in the treatment of HIV-1 infection in combination with other antiretrovirals (ARVs). These non-nucleoside reverse transcriptase inhibitors (NNRTIs) are essential components of highly active antiretroviral therapy (HAART) against HIV-1 infections. NNRTIs bind to an allosteric site of reverse transcriptase, resulting in inhibition of the enzyme activity. Implications of oxidative stress during long-term antiretroviral therapy with NNRTIs are also reported, thus complicating the existing scenario further [9].

Mechanisms of viral pathology reveal that a redox imbalance in the cells, as a result of oxidative stress, causes dysregulation in the levels of glutathione (GSH) [2,5–7]. GSH in the reduced form is the major intracellular redox buffer, exerting its effect directly or indirectly regulating antimicrobial activity in immune cells [6].

GSH-enhancing agents elevate the cellular immune response while antagonizing the humoral response. This is accomplished mainly by favoring cytokine expression profiles of TH-1 CD4+ cells and promoting the functions of NK cells. Another explanation for the rising incidence of *M. tb* among individuals with an HIV infection is due to decreased levels of GSH, which impairs immune cell function [10]. Our lab has previously demonstrated that rGSH directly controls *M. tb* infection through natural killer cells and T cells [2,11]. A therapeutic limitation to GSH is that a high dose is necessary to achieve the desired effect. GSH is known to have a short plasma half-life along with its inability to cross the cell membrane. Instead, GSH needs to be broken down into amino acids and resynthesized to GSH intracellularly—a process often impaired during viral infections [12]. Earlier clinical findings report that supplementation with liposomal glutathione (L-GSH) restored redox homeostasis, induced a cytokine balance, and improved immune responses against *M. tuberculosis* infection [13,14]. To overcome its biochemical and pharmacokinetic limitations, pro-GSH molecules have been proposed to restore or increase GSH levels for easier membrane transportation or constitute a source of thiols for GSH synthesis [15–17].

On the other hand, antiretroviral therapy (ARV) has reduced AIDS-related morbidity and mortality. However, several drawbacks in the current therapy still exist. Failure to target macrophages and dendritic cells that are involved in the transmission of the virus to CD4+ TH cells (helper T lymphocytes) increases the risk of relapse [18]. Among the various approaches to treat HIV infection, strategies targeting viral reservoirs are highly attractive. In this context, the development of delivery systems that can target monocytes/macrophages intracellularly is crucial. Liposomes are the most widely investigated delivery system for phagocyte-targeted therapies, providing advantages such as low immunogenicity, biocompatibility, cell specificity, and drug protection [19]. Lipid-based nanocarriers are reported to hold great promise, with 15 commercial nano lipid medicines as liposomes or lipid nanoparticles, which includes AmBisome^®^, DaunoXome^®^, Amphotec^®^, Fungizone^®^, Diprivan^®^, Estrasorb^®^ Mepact^®^, Myocet^®^, Visudyne^®^, DepoCyt^®^, DepoDur^®^, Doxil^®^,Inflexal^®^ V, Marqibo^®^, Abelcet^®^ [20–22]. A recent inclusion is the Pfizer/BioNTech Moderna COVID-19 vaccines used for prophylaxis of severe acute respiratory syndrome coronavirus 2 (SARS-CoV-2) infection. One of the main reasons for their well-acceptance is because of their structural units, which are generally recognized as safe (GRAS) [23].

The combinational administration of GSH and ARV agents, such as efavirenz, during HIV infection treatment is among one of the most underexplored areas to the best of our knowledge. GSH supplements have been considered useful because they allow patients to alleviate toxic side effects, decrease multidrug resistance (MDR), and provide protection against opportunistic mycobacterial infection.

After administration, liposomes are avidly removed from the circulation by the macrophages of the reticuloendothelial system (RES) or mononuclear phagocyte system (MPS) [24]. Apparently, this propensity to be taken up by the macrophages is advantageous in the treatment of diseases such as AIDS, where macrophages harbor infectious HIV [24,25]. This can be achieved by varying lipid composition to control physicochemical properties such as size and charge [19]. The size of liposomes ranging from 50 to 800 nm in diameter and composed of different types of lipids with neutral, positive, or negative charges have been shown to influence the drug uptake by macrophages [19,26]. Various examples of therapeutic applications using monocyte/macrophage-targeted liposomes are reported elsewhere. Among them is dideoxycytidine-5-triphosphate for HIV [19]. With efavirenz being a lipophilic molecule and glutathione a hydrophilic molecule, delivery of EFA + GSH can be achieved via liposomes. Liposomes are versatile carriers to encapsulate either/and a hydrophilic or lipophilic molecule, therefore suitable for co-delivery of one or two molecules. Evidence obtained by in vitro studies indicates that short-term treatments with clinically relevant concentrations of EFA reduce cellular proliferation and/or compromise cell viability [9].

Since prevention is becoming a key strategy to eliminate HIV, research in these directions clearly warrants dual drug delivery of antiretrovirals and antioxidants.

We hypothesize that delivery of EFA and GSH formulated as liposomal carriers could promote site-specific drug delivery, eliminate viral accumulation, and provide cytoprotective effects in non-infected macrophages (THP-1 cells). Hence, the goal of the present work is to investigate the intracellular uptake of EFA-loaded liposome (with and without GSH) by human monocytic leukemia cells (THP-1 cells) and examine cell viability and ROS scavenging activity.

## 2. Materials and Methods

### 2.1. Materials

Efavirenz was obtained from VWR, USA. Dimyristoylphosphatidylcholine (DMPC) and distearoyl-phosphatidylcholine (DSPC) were purchased from Avanti Polar Lipids (Alabaster, AL, USA). Phospholipon 90H (PL-90H) was obtained from Lipoid LLC (Newark, NJ, USA). Cholesterol, n-dodecane, Roswell Park Memorial Institute media (RPMI), AB serum, Phorbol 12- myristate 13-acetate (PMA), poly-lysine and 10% fetal bovine serum (FBS) were purchased from Sigma Aldrich (St. Louis, MO, USA). Inertsil, ODS-3 was purchased from GL sciences, Torrance, CA, USA. Paraformaldehyde (PFA) was purchased from EMD Chemicals, MA, USA. DAPI fluoromount was obtained from Southern Biotech, AL, USA. Rhodamine 123 was purchased from Marker Gene Technologies, Oregon, USA. All the organic solvents were HPLC grade and obtained from Fisher Scientific (Pittsburgh, PA, USA). CellTiter 96^®^ Aqueous One Solution Reagent was purchased from Promega, Madison, WI, USA. CellROX^®^ Deep Red Reagent was obtained from (CAT 10422) Molecular Probes, Eugene, OR, USA.

### 2.2. Methodology

#### 2.2.1. Formulation of Liposomes

Efavirenz liposomes: Liposomes loaded with efavirenz were prepared as per the composition shown in Table 1 using thin-film hydration method. Initial formulations were prepared using different lipids with and without tween-80. Briefly, lipid phase composed of lipids and cholesterol was prepared using chloroform or CHCl_3_: MeOH (2:1 *v*/*v*) in a dry round bottom flask. Organic solvent was removed by a vacuum evaporator (Rotavapor-R, W.Büchi, Flawil Schweiz) above the respective lipid transition temperature (DMPC 23 °C; DSPC 55 °C; SPC 51 °C and DMPC/DMPG 23 °C). The deposited lipid film was further stored overnight in the desiccator under vacuum for the complete removal of organic solvent. The film was hydrated with an appropriate volume of phosphate buffer pH 6.8 by rotation for 1–2 h at a temperature above 23 °C. Small unilamellar (SUV) liposomes were obtained by subjecting the dispersions to probe sonication for 2 min using cooling pads. Control liposomes were prepared similarly without efavirenz. Finally, the liposomal dispersions prepared were stored at room temperature for 2 h to anneal any structural defects. To separate the non-encapsulated drug from liposomal suspension, Sephadex^®^ G-50 fine (1 cm × 20 cm) column equilibrated with 150 mM NaCl solution was used. Opalescent liposome fractions collected were used to determine the drug concentration.

Efavirenz, being a lipophilic molecule, was included in the lipid phase; GSH was added in the aqueous phase during the lipid film hydration to prepare glutathione liposomes; liposomal formulation of EFA + GSH combination consisted of EFA and GSH in respective phases in the formulation.

#### 2.2.2. Characterization of Liposomes

Particle size and zeta potential: Liposomal dispersion of EFA liposomes was used to determine the particle size (Z-average mean size and polydispersity index) and ζ—potential using Nano ZS90, Malvern, Malvern Instruments Ltd., Worcestershire, UK) using dynamic light scattering (DLS) after appropriate dilution (1:10 with water). An aliquot of EFA liposomal formulation was further diluted (1:100) and filled into electrophoretic cell. Zeta potential was determined from the electrophoretic mobility of the sample.

Encapsulation efficiency (EE): To measure the amount of efavirenz encapsulated, liposomal suspension of EFA and EFA + GSH was used. Centrifugal ultrafiltration was performed using Nanosep 300 K (MWCO 300 kDa) and 100 K (MWCO 100 kDa). A 0.5-mL aliquot of liposomes was loaded and centrifuged at 5 °C for 5 min at 14,000 rpm. The filtrate was collected, and the content of EFA and GSH was analyzed using HPLC [27,28]. Encapsulation efficiency (%) was calculated using the following Equation (1).

Encapsulation Efficiency (EE):

(1) $$\text{EE}\%=\frac{\text{Total amount of drug }-\text{ amount of drug unentrapped}}{\text{Total amount of drug}}\times 100$$

### 2.3. HPLC Analysis

Liquid chromatography was carried out by Agilent 1260 Infinity LC system consisting of a thermostated autosampler, a binary pump, a degasser, and a thermostated column compartment. Analytes were monitored using a poly diode array detector (PDA). Open lab was the software package used for system control and data acquisition.

#### 2.3.1. Chromatographic System for Efavirenz Analysis

Separation was performed using Inertsil, ODS-3 (5 micron, 250 × 4.6 mm) analytical column. A reversed-phase isocratic elution was achieved using a mobile phase consisting of 75% solvent A (acetonitrile) and 25% solvent B (Milli-Q water; pH adjusted to ±3.2 using 1% formic acid). All solvents were degassed prior to use. EFA was detected at 247 nm at a flow rate of 1 mL/min at ambient temperature, the run time was 10 min, and the injection volume was 10 μL. Stock solutions of EFA were prepared using the mobile phase composition to achieve a final concentration of 0.01 mg/mL. Method was validated for linearity (r^2^ = 0.9997; y = 16.519x − 1.989).

#### 2.3.2. Chromatographic System for Glutathione Analysis

Separation was performed using XBridge C-18 Waters column (5 μm× 4.6 mm× 150 mm). Isocratic elution was achieved using a mobile phase consisting of 10% solvent A (methanol) and 90% solvent B (Milli-Q water; pH adjusted to ±2 using 0.1% TFA). All solvents were degassed prior to use GSH was detected at 210 nm at a flow rate of 1 mL/min at ambient temperature, run time was 10 min, and the injection volume was 20 μL. Stock solutions of GSH were prepared using the mobile phase composition to achieve a final concentration of 0.01 mg/mL. Method was validated for linearity (r^2^ = 0.9883; y = 10.341x – 91.461).

### 2.4. Intracellular Uptake of EFA Liposomes in Mo/Mac Cells (THP-1 Cells)

#### 2.4.1. THP-1 Cell Culture

Human monocytic leukemia cells (THP-1 cell line) were cultured in RPMI medium with 10% fetal bovine serum at 37 °C and 5% CO_2_. After resuspension, THP-1 cells (2 × 10^5^/well) were distributed in poly-lysine coated 24-well tissue culture plates. Differentiation of THP-1 cells to macrophages was achieved by adding PMA (Phorbol 12-myristate 13-acetate) at a concentration of 10 ng/mL and incubated for 24 h.

#### 2.4.2. Quantification of Efavirenz Intracellular Uptake

To assess the intracellular targeting capacity of lipid carriers, differentiated macrophages were incubated with EFA loaded liposomes and aqueous EFA dispersion (50 μM to 400 μM) for 24 h at 37 °C with 5% CO_2_. THP-1 cells were washed in sterile 1X PBS. To determine the concentration of EFA in the cell lysate, 100 μL of methanol was added to 100 μL of cell lysate sample, vortexed, centrifuged at 1500 rpm for 15 min, and the filtered supernatant was used to analyze the concentration of EFA using HPLC.

#### 2.4.3. Rhodamine 123 (R123) Loaded EFA Liposomes and EFA Aqueous Dispersions

Liposomes loaded with Rhodamine 123, a fluorescent dye in the lipid phase (0.04% *w*/*w* of lipid) and aqueous dispersion of pure EFA prepared with R123 was used to compare the uptake using confocal microscopy.

#### 2.4.4. Confocal Laser Scanning Microscopy (CLSM)

To perform imaging studies, sterilized cover glass (coated with 0.001% poly-lysine) was placed in the cell culture plates, and cells were cultured on the cover glass using RPMI fresh media supplemented with 10% FBS. When the cells were ready to be used, rhodamine123 loaded preparations were added and incubated for 24 h. Then the cells were washed with ice-cold PBS and fixed with PFA (3.8% paraformaldehyde) for 30 min at room temperature. Further, the cells were washed with PBS 3 times (5 min per time). Cover glass was inverted and mounted onto slides with mounting media containing DAPI stain to visualize cell nuclei, further sealed cover glass by clear nail polish stored in dark till imaging. Images were captured by the Leica TCS SPE-II system (Leica Microsystems Italia, Milan, Italy). Uptake studies of fluorescent liposomes by differentiated macrophages were carried out by CLSM. Selected wavelengths were 365–375 nm (DAPI, blue) and 450–490 nm (R123, green).

### 2.5. MTS Cytotoxicity Assay

Cell viability and in vitro cytotoxicity was performed using MTS assay, as it offers several advantages over other cytotoxicity tests such as MTT [29]. Intensity of the color varies based on the amount of formazan and is proportional to the number of viable cells; reduction of this intensity is indicative of cellular damage. To perform this assay, 20 μL of CellTiter 96^®^ Aqueous One Solution Reagent, which is composed of the novel tetrazolium compound MTS and an electron coupling reagent phenazine methosulfate (PMS), was added to each well containing cells treated with EFA. After 3 h in culture, the cell viability was determined by measuring the absorbance of a coloured, water-soluble formazan at 490 nm using a microplate reader. Results are expressed as a percentage of viability in control cells incubated in medium alone

### 2.6. Measurement of ROS-Induced Fluorescence by Flow Cytometry

The cell-permeant CellROX red reagent is non-fluorescent in a reduced state and produces bright near-infrared fluorescence upon oxidation. The resulting fluorescence was measured using flow cytometry with absorption/emission wavelength at ~644/665 nm. In addition to allowing ROS detection in live cells, the signal was retained after formaldehyde fixation. EFA preparations (pure EFA and liposomal EFA with and without GSH) were added and incubated for 24 h at 37 °C with 5% CO_2_. After 24 h, the cells were washed with ice cold PBS and fixed with PFA (3.8% paraformaldehyde) per well for 30 min at room temperature. Further, the cells were washed with PBS for 3 times (5 min per time). Thereafter, the cells were centrifuged at 1000 RPM; the pellet was re-suspended with PBS and stored on ice for immediate FACS analysis (BD Accuri^™^ C6 P flow cytometer, BD Biosciences, San Jose, CA, USA). Fluorescence intensity of cellROX was measured in FL-4 at a wavelength of 640 nm. For each measurement, a total number of 50,000 events were recorded and analyzed using CFlow Plus^®^ software.

### 2.7. Statistical Analysis

Data are presented as mean ± standard deviation. ‘n’ represents the number of independent experiments. Differences were tested for significance using two-way ANOVA, one way ANOVA and unpaired *t*-test. Tukey’s and Bonferroni’s multiple comparisons test were used as a post hoc test. *p* < 0.05 was considered to be significant.

## 3. Results

### 3.1. Formulation and Characterization of Liposomes

Liposomal formulations were prepared initially to encapsulate efavirenz alone (without GSH) using different lipids/ lipid mixtures along with cholesterol (Table 1) and water as hydration media. As depicted in Table 1, we observed that liposomal suspension prepared using a single lipid led to coagulation within 24 h of storage, whereas formulations prepared using lipid mixture of DMPC/DMPG-Na along with cholesterol, hydrated with phosphate buffer pH 6.8 were stable. Hence, this composition was selected to prepare EFA liposomes with and without GSH, which is illustrated in Table 2.

On the other hand, GSH, an endogenous tripeptide, is a water-soluble molecule with a molecular weight of 307.32 Da that was to be encapsulated within the aqueous phase. Liposomes are able to encapsulate: (a) hydrophobic molecules such as efavirenz in the bilayer membrane and (b) hydrophilic compounds such as glutathione in the aqueous internal cavity.

Hence in this present work, lipid mixtures (DMPC and DMPG) were used to encapsulate hydrophobic EFA, which led to % EE greater than 90% facilitating its inclusion into the lipid bilayers and hydrophilic GSH (% EE of 43%) within the aqueous core during the formation of the liposomes.

Particle size reported as the Z-average diameter was about 120 nm to 205 nm without and with GSH, respectively, whereas polydispersity index value <0.3 indicated that liposomal vesicles were unimodal, revealing that sonication led to the formation of a homogeneous population.

Negative surface charge on the liposomes as indicated by the zeta potential values were greater than −50 mV leading to stable suspensions with reduced aggregation. The negative surface charge could be due to the polar head group of phospholipid and hydroxyl groups of cholesterol [30].

### 3.2. Intracellular Uptake by Macrophages

**Figure 1.**
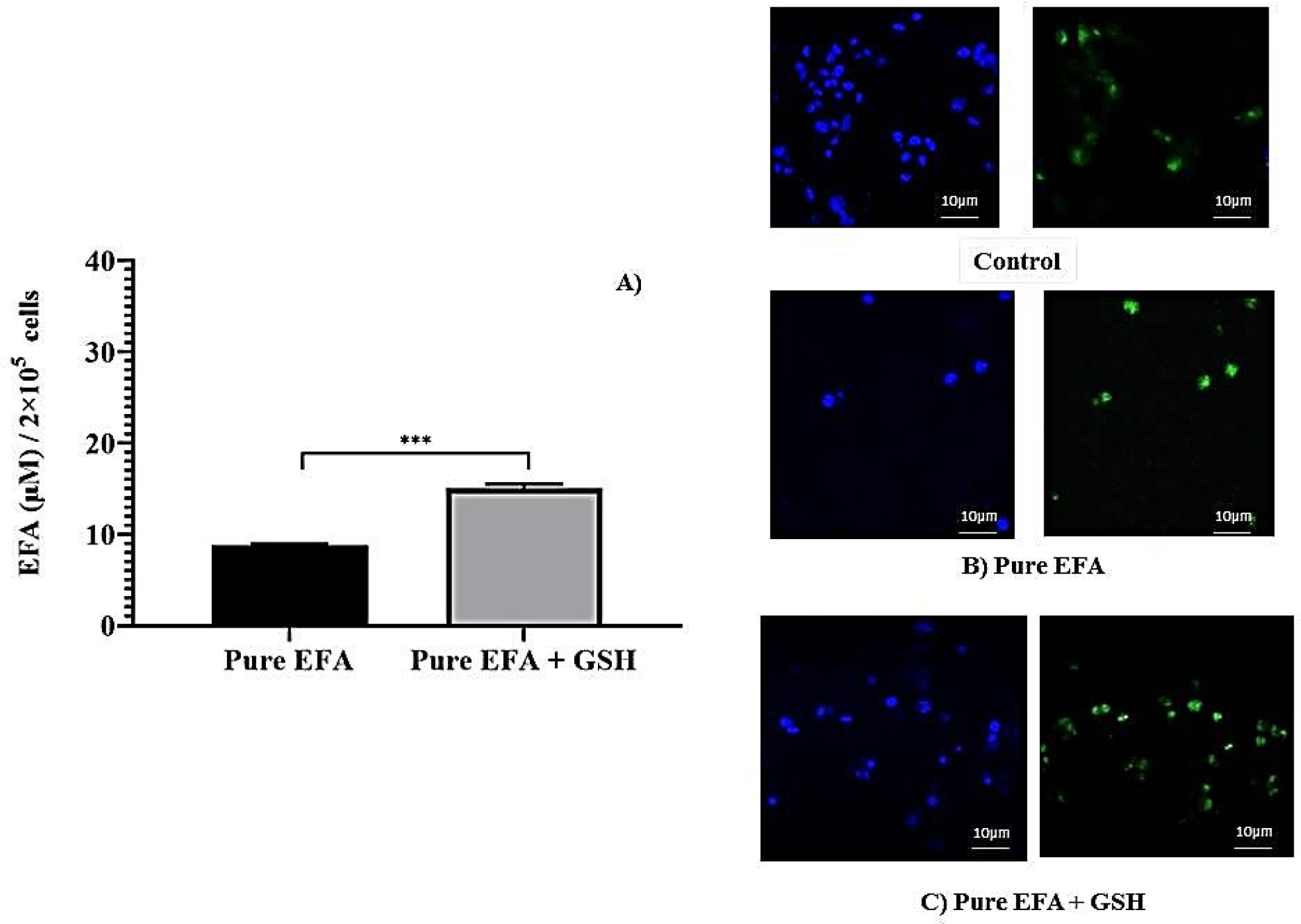
(**A**) Comparison of intracellular lysate concentrations of pure EFA with and without GSH (*** indicate *p* < 0.001). Values are means ± S.D. of three independent experiments. (**B**,**C**) represent the uptake of rhodamine123-containing pure EFA without and with GSH by the THP-1 monocyte/macrophage cells respectively. Cells were incubated with sample preparation for 24 h at 37 °C. Fluorescent images were acquired at ×40 original magnification. Nucleus is stained with DAPI (blue); R123-loaded EFA shown as green fluorescence.

**Figure 2.**
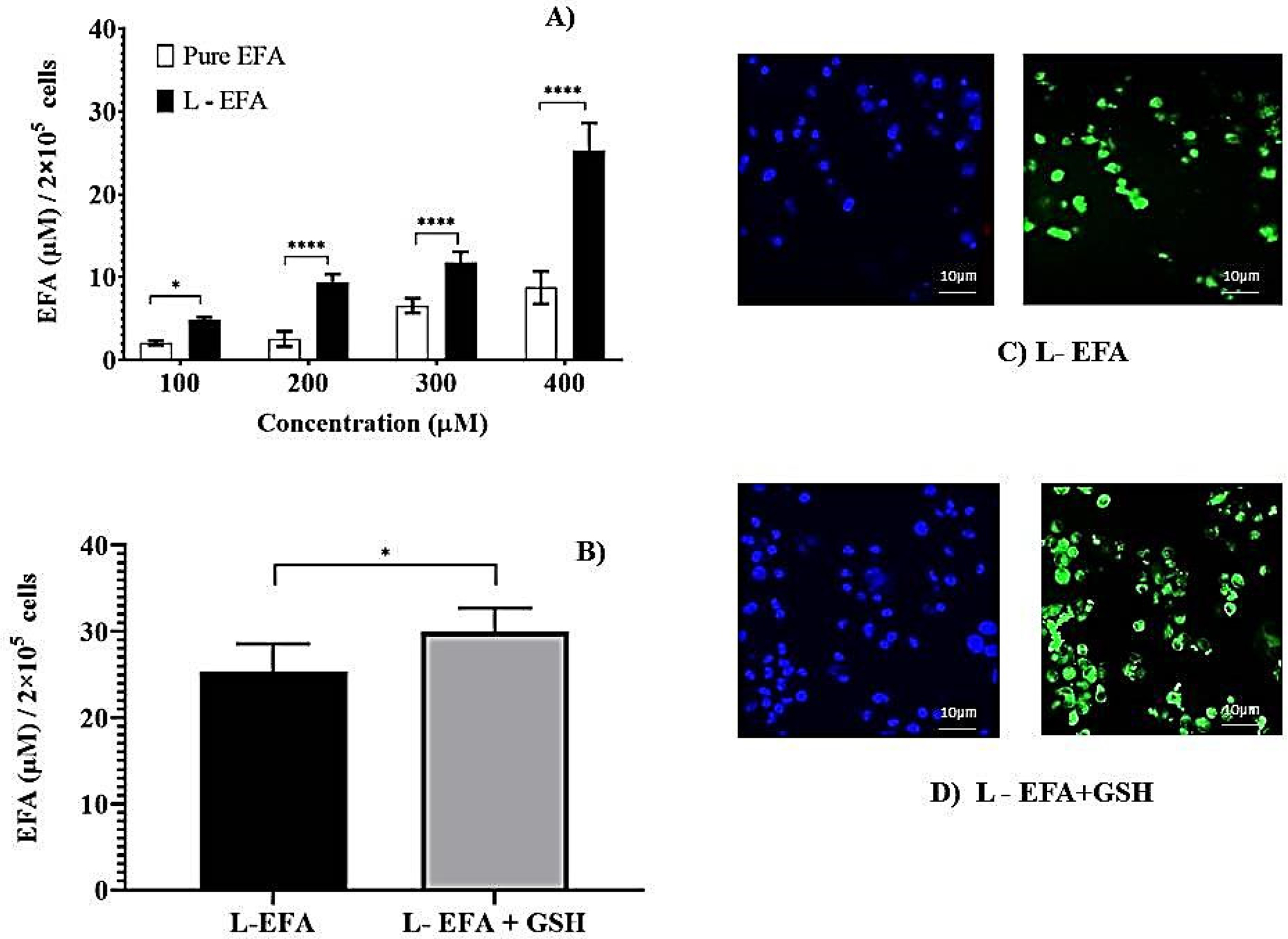
(**A**) Intracellular lysate concentrations of pure EFA and liposomal EFA after 24 h at 37 °C incubation of THP-1 monocyte/macrophage cells with liposomal EFA. (**A**) Intracellular lysate concentrations of EFA with varying concentrations of pure (pure EFA) and liposomal EFA (L-EFA), significant difference between uptake of pure EFA and L-EFA was observed at 200, 300 and 400 μM (**** indicate *p* < 0.0001). (**B**) Significant difference between uptake of liposomal EFA with and without GSH was observed (* indicate *p* < 0.05). Values are means ± S.D. of three independent experiments (**C**,**D**) represent the uptake of rhodamine 123-containing liposomal EFA with and without GSH by the THP-1 monocyte/macrophage cells. Cells were incubated with sample preparation for 24 h at 37 °C. Fluorescent images were acquired at ×40 original magnification. Nucleus is stained with DAPI (blue); R123-loaded EFA liposomes shown as green fluorescence.

To evaluate the influence of glutathione on the EFA uptake, cells were treated with a pure form of efavirenz with and without GSH, (Figure 1). Later, a liposomal form of EFA and GSH were examined (Figure 2). It was observed that the addition of GSH along with EFA demonstrated an almost two-fold increase in the intracellular concentration of pure EFA in comparison to pure EFA alone Figure 1A. To further confirm the uptake, confocal images were captured, which revealed that the viability was improved, as evidenced by an increase in the number of cells (Figure 1B,C).

Further, the incorporation of EFA into liposomes was investigated and compared with pure EFA at various concentrations. As represented in Figure 2A, the initial dose-escalation study was carried out using 100 μM to 400 μM of EFA in the pure form and liposomal form. After 24 h of incubation, intracellular lysate concentrations revealed a significantly higher amount of EFA taken up by the cells from EFA liposomes compared to pure EFA. A dose-dependent increase in the uptake of EFA was observed. Nevertheless, at all concentrations evaluated, we observed that encapsulation of EFA into the liposomes increased its uptake by the macrophages. The concentration of 400 μM resulted in the highest uptake when liposome loaded EFA was administered, which was selected for further evaluation.

Further, the influence of the immunomodulatory agent GSH on the uptake of EFA encapsulated into liposomes was examined. Formulation composed of EFA + GSH (F2) was incubated with the THP-1 cells. As depicted in Figure 2B, the inclusion of GSH into EFA liposomes led to a 1.2-fold increase in EFA uptake in comparison to EFA liposomes at the end of 24 h of cellular uptake study.

Thus loading EFA into liposomes influenced higher uptake, which was augmented in the presence of GSH (100 μM). Various other studies have reported that lipid composition and charge can affect the cellular uptake of drug encapsulated liposomes. Liposomes having long saturated fatty acyl phospholipid along with cholesterol (50% molar ratio) displayed the best stability in the intra-macrophagic compartment [31]. Monocyte/macrophage cellular uptake of tritium labelled stavudine (d4T) liposomes revealed that the presence of sphingomyelin decreased the uptake of d4T, and the presence of negative charge was more effective [24]. In the present investigation, negatively charged EFA + GSH liposomes were found to have improved uptake.

To supplement the quantitative measurement of cell uptake, images of the formulations labelled with Rhodamine 123 were captured using confocal microscopy. After 24 h incubation, the microscopic images displayed intact cells, and the respective fluorescent images visualized the uptake of RH123 labelled liposomes by these cells. The nuclei were stained with DAPI (blue). As noticed in Figure 2C,D, the images reveal that a higher amount of EFA from liposomes was internalized by the THP-1 Mo/Mac cells. In comparison to the pure form of EFA, liposomal EFA demonstrated an enhanced fluorescent Rhodamine uptake by the cells, which is clearly evidence of intracellular localization of drug, further intensified in the presence of GSH.

### 3.3. Cytotoxicity Study

**Figure 3.**
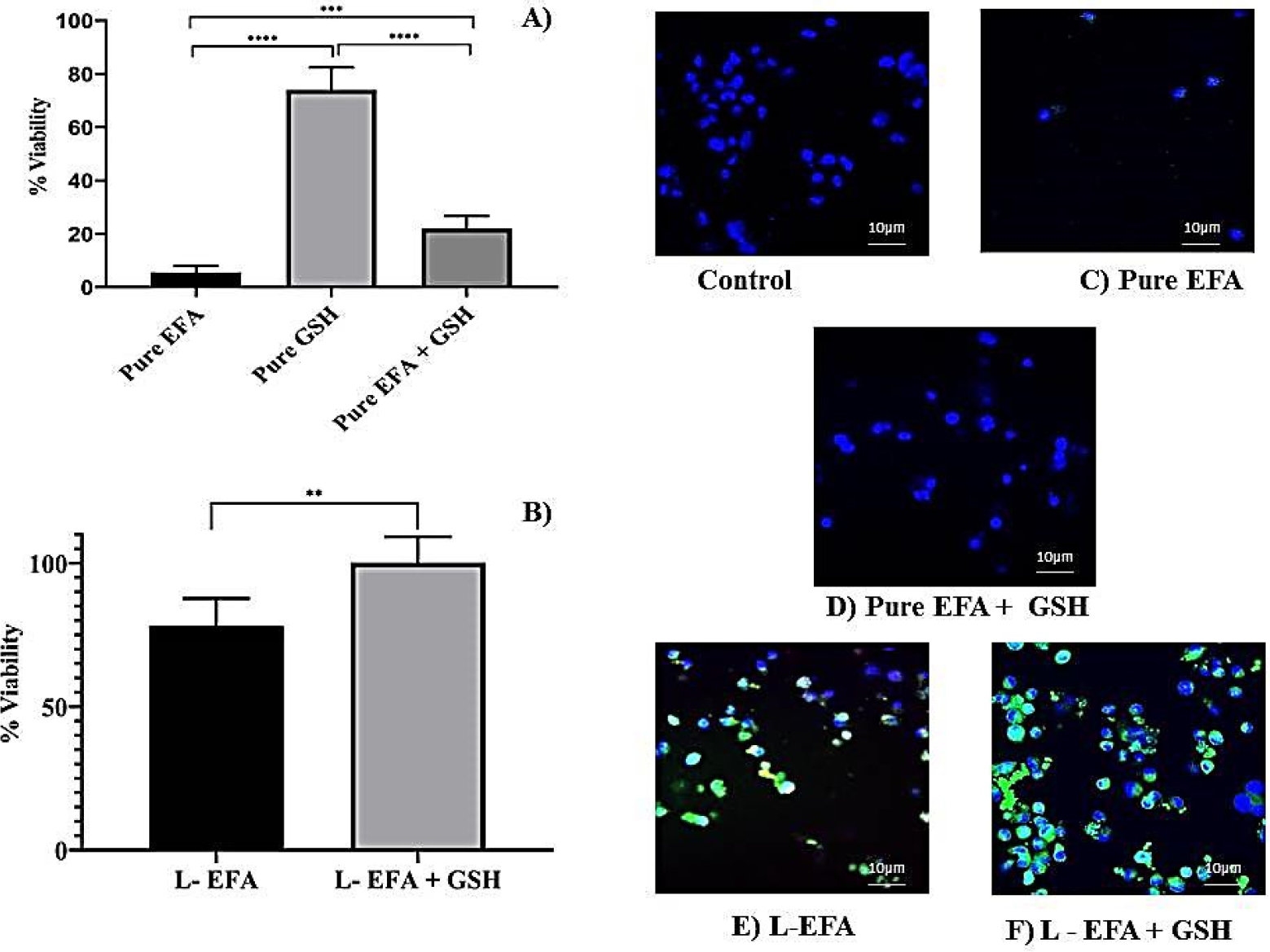
(**A**) Influence of EFA on viability of THP-1 monocyte/macrophage cells treated with pure EFA with and without GSH after 24 h incubation; (**B**) Influence of liposomal loaded EFA with and without GSH on viability of THP-1 monocyte/macrophage cells; (**C**–**F**) represent merged fluorescent images acquired at × 40 original magnification. Control indicates untreated cells, whereas cells were treated with pure EFA without and with GSH (**C**,**D**); liposomal EFA without and with GSH (**E**,**F**). Nucleus is stained with DAPI (blue); R123-loaded EFA liposomes shown as green fluorescence (**** indicate *p* < 0.0001; *** indicate *p* < 0.001 and ** indicate *p* < 0.01). Values are means ± S.D. of six independent experiments.

The effect of EFA and GSH on the viability of THP-1 cells as assessed by the MTS method is illustrated in Figure 3. An aqueous dispersion of pure EFA was prepared using 1% sodium CMC as a suspending agent. Using the data obtained from cell uptake studies, we tested the viability at a selected concentration of EFA (400 μM) to assess the cytotoxicity potential of the liposome formulation in comparison to the pure form of EFA.

MTS results indicated that EFA formulated in liposomes significantly increased the cell viability up to 78% of untreated cells in comparison to only 5 % with pure EFA (Figure 3A,B).

The potentiality of pure GSH, when incubated alone or co-administered with pure EFA in THP-1 cells, is depicted in Figure 3A,B. In the present work, it was observed that co-administration of GSH enhanced the viability of EFA by 4-fold despite that the viability with GSH alone was >70%. As portrayed in Figure 3C,D, the presence of GSH improved the viability of THP-1 cells in the presence of EFA to 21.9% from 5.5% (about 4-fold). Further, delivery of liposome-encapsulated EFA + GSH signifies the influence of glutathione, as the viability was 100% relative to control cells. MTS results clearly demonstrated that GSH improved the viability of THP-1 non-infected cells. Overall, MTS assay provides strong evidence that EFA is encapsulated into liposomes and, when co-delivered with GSH, significantly increases the cell viability by 15.6-fold and 20-fold, respectively, in comparison to pure EFA. Supportive to the colorimetric estimation by MTS assay, confocal images displayed an increase in the number of cells and increased uptake (overlay) of R123 labelled liposomes (Figure 3E,F), demonstrating the biocompatibility and non-toxicity of liposomes. This was further confirmed by the elimination of cytotoxicity of EFA at a concentration of 400 μM when encapsulated into liposomes.

### 3.4. Measurement of ROS-Induced Fluorescence by Flow Cytometry

**Figure 4.**
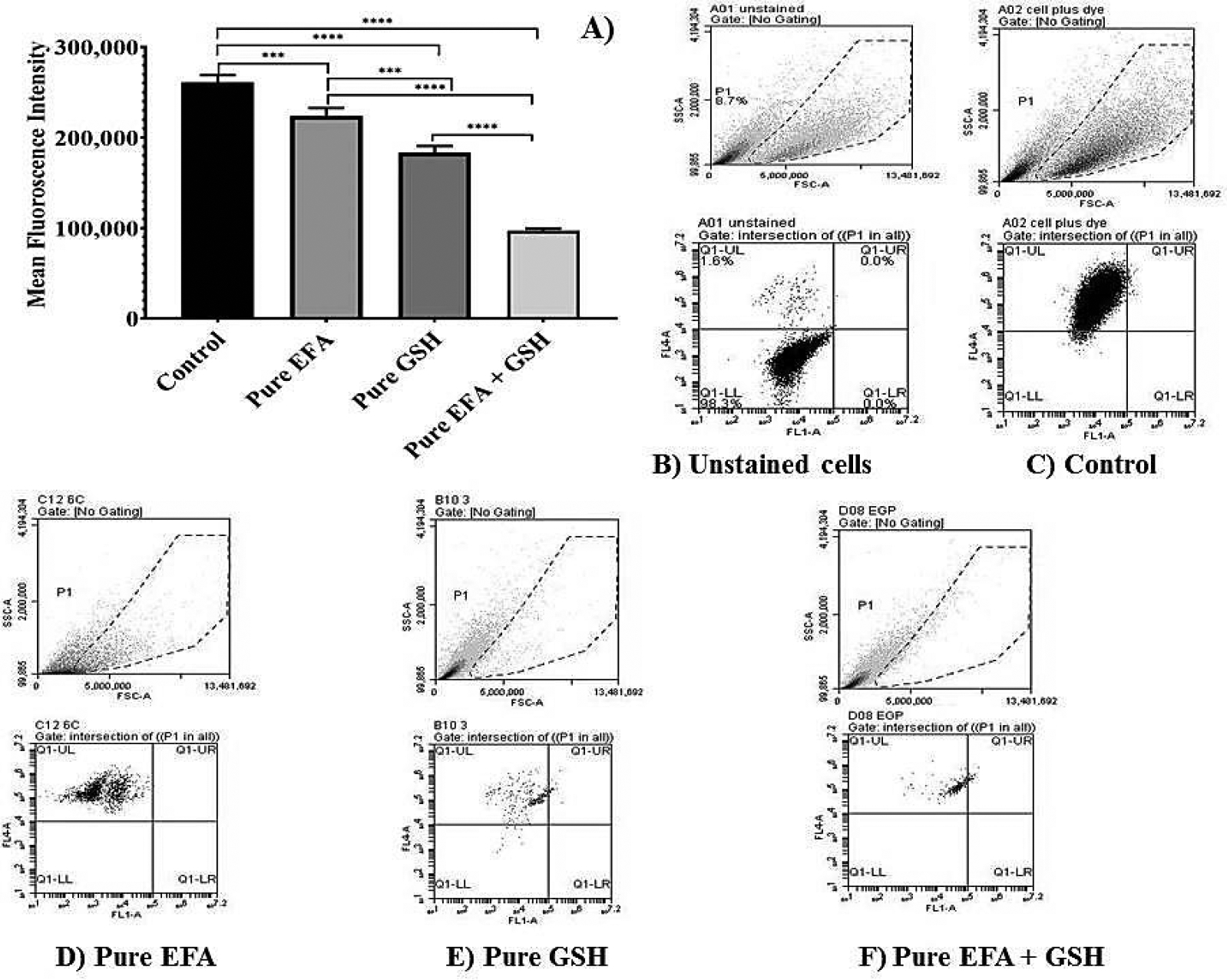
Flow cytometry analysis depicting the measurement of reduction in mean fluorescence intensity (MFI) of PMA-induced oxidative stress in THP-1 monocyte/macrophage cells. (**A**) Dot plots representing the data of MFI reduction in cells treated with pure efavirenz (EFA) with and without GSH in comparison with pure GSH (**** indicates *p* < 0.0001; *** indicates *p* < 0.001).Values are means ± S.D. of three independent experiments. (**B**–**F**) represent dot plot of forward scatter to cellROX-derived red fluorescence showing the cellROX gate (gated events) and the below plots represent the dot plot depicting ROS-dependent cellROX fluorescence when cells treated with pure EFA with and without GSH. Untreated cells with no cellROX dye (**B**); untreated cells with cellROX dye (control—(**C**)).

**Figure 5.**
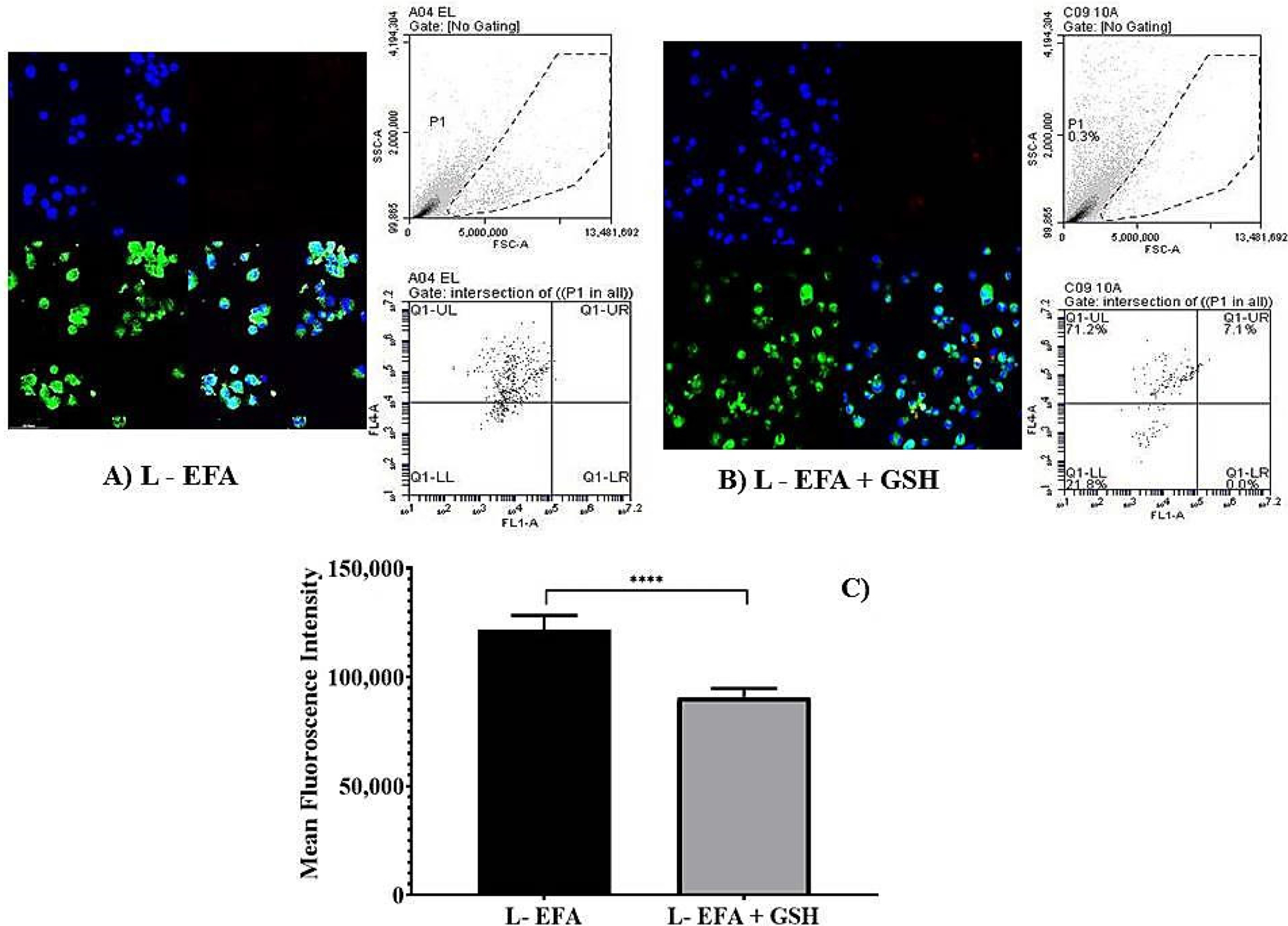
Flow cytometry analysis depicting the measurement of reduction in mean fluorescence intensity (MFI) of PMA-induced oxidative stress in of THP-1 monocyte/macrophage cells. (**A**,**B**) represent dot plot of forward scatter to cellROX-derived red fluorescence showing the cellROX gate (gated events) and the below plots represent the dot plot depicting ROS-dependent cellROX fluorescence when cells treated with pure EFA with and without GSH. Confocal fluorescent images acquired at × 40 original magnification from cells treated with Liposomal EFA without GSH (**A**) and with GSH (**B**). Nucleus is stained with DAPI (blue); R123-loaded EFA liposomes shown as green fluorescence and oxidative stress is shown as red fluorescence. (**C**) Represents the data of MFI reduction in cells treated with liposome loaded efavirenz (EFA) with and with GSH in comparison; (**** indicate *p* < 0.00001) significant difference of liposomal EFA and liposomal EFA + GSH. Values are means ± S.D. of three independent experiments.

The results of the flow cytometry analysis using THP-1 cells treated with pure forms EFA and GSH alone and in combination is depicted in Figure 4, and when encapsulated into liposomal carriers is shown in Figure 5. Comparison of mean fluorescence data when cells are incubated with pure EFA and pure GSH and a combination of both is exhibited in Figure 4A. As observed, a strong fluorescent signal of CellROX was visible in the control cells due to PMA-induced ROS generation (Figure 4B,C). In the present study, an increase in total ROS was observed, indicating an increase in the oxidative stress in response to the treatment with EFA (Figure 4D) as reported in earlier studies also [9,32,33].

Forward scatter (FSC) vs. side scatter (SSC) dot plots were used to visualize the expression of fluorescence of the cells. Dot plots obtained from the flow cytometric analysis demonstrated that ROS-induced fluorescence was reduced by 1.2 fold in comparison with pure EFA, and was further reduced by 2.3 fold in the presence of GSH (Figure 4D,F).

However, to further evaluate the ability of liposomal systems to reduce the ROS-dependent cellROX fluorescence, THP-1 cells were also incubated with the EFA liposomes (Figure 5A) and EFA + GSH liposomes (Figure 5B) for 24 h. The overlay image (Figure 5A,B) indicate that the combination of GSH with EFA was more active than the singly loaded EFA liposomes and the pure EFA as well. Hence, EFA-loaded liposomes effectively protected the oxidative damage by 1.8 fold relative to pure EFA. Data obtained from flow cytometric analysis reveal that EFA, when encapsulated into liposomes, could effectively quench the intracellular accumulation of ROS, exhibiting a synergistic effect in combination with GSH (2.5 fold) as displayed in Figure 5C.

## 4. Discussion

With the inception of highly active antiretroviral therapy (HAART), the quality of life of HIV-infected patients has seen a major transition and has increased life expectancy. The combination of drugs efficiently suppresses an HIV viral load, thereby prolonging the life of HIV/AIDS patients almost to one of the general population. However, HIV infection leads to pronounced oxidative stress through various mechanisms by which the virus triggers ROS production [34]. Virus triggered ROS production is a strong modulator of the immune system leading to opportunistic infections [34]. Thus, such effects need to be controlled and targeted for immune suppression of viral replication. In a wide range of disease states characterized by increased oxidative stress, antioxidants have been demonstrated to boost immune function as reported in human intervention studies [35]. Furthermore, immune modulators, which are potential candidates in the prevention and treatment of HIV/AIDS, are thoroughly reviewed and reported elsewhere [36].

Research findings have demonstrated that an alteration of the intracellular redox balance characterizes several viral infections and the progression of viral-induced diseases [5]. In case of HIV, it has been suggested that increased levels of inflammatory cytokines can induce both depletion of GSH and oxidative stress [34,37–39]. GSH, being the most powerful antioxidant, puts the cell at risk for oxidant damage when it is deficient. It has been observed that a wide range of pathologies, including cancer, and many viral infections, including HIV-1, leads to an imbalance in GSH levels, and various reports have provided evidence for the use of GSH molecules being able to replenish intracellular GSH levels during antiviral therapy [2,11,34]. While the combined use of antioxidants and chemotherapeutic agents has been explored, the interactive effects of using ARVs and AOs are very limited.

The drug of interest in the present investigation was efavirenz (EFA), which remains the ARV of choice when it comes to the treatment of individuals co-infected with mycobacterial infections. Efavirenz is known to exhibit similar behaviour to so-called brick dust drugs due to its high melting point and stable crystalline structure in which strong intermolecular bonds significantly limit the dissolution and bioavailability of their pharmaceutical forms [40]. Poor solubilization of efavirenz, a benzoxazinone compound with 40 to 45% of oral bioavailability, obscures its therapeutic functionality. To enhance the therapy of HIV infection, lipid-based oral drug delivery systems of such poorly soluble drugs are under investigation. Nevertheless, various other lipid carriers of EFA are reported by several authors [41–45]. However, liposomal delivery to achieve effective drug concentrations in viral reservoirs has gained importance in the last few decades, yet EFA delivery by way of liposomes is limited. Furthermore, its co-delivery along with GSH is not reported so far. Another molecule, nevirapine belonging to the group of NNRTI, has been reported to be loaded into liposomal formulations to improve targeted delivery to select compartments in the cells, thereby alleviating systemic toxic side effects [46].

By application of film hydration procedure, liposomes incorporating EFA with and without GSH were prepared to obtain encapsulation efficiency up to the range of 90%. The encapsulation efficiency of GSH was less prominent, which could mean that a substantial amount of GSH was present in the aqueous core. Furthermore, the polydispersity index less than 0.3 indicated a narrow size distribution and homogenous distribution of colloidal particles, hence a good quality for all the formulations. Furthermore, a large negative zeta potential further enhanced the stability of vesicular suspension by simple electrostatic stabilisation with negativity being imparted by phospholipid and cholesterol. The presence of GSH in liposomes loaded with EFA did not influence the physicochemical properties when compared with liposomes loaded with EFA alone (Table 2). Earlier, encapsulation of cytochrome C, a hydrophilic protein was, along with lipophilic vitamin E, developed to obtain more efficient drug encapsulations and examine their release using the same lipid mixture [47], whereas using a single lipid such as phospholipon 90H, lipophilic resveratrol was encapsulated along with a hydrophilic vitamin C to investigate the ameliorative antioxidant effect [48].

HIV-1 infection harbors the virus in latent reservoirs such as macrophages, one of the key targets of HIV-1 infected patients. Macrophages, commonly referred to as virus reservoirs sites, are not killed by the HIV infection, thus aiding the virus to reproduce and supply the body with new virus [49]. It is well known that macrophages play a crucial role in the host defense against HIV-1 infection, as they produce multiple intracellular HIV restriction factors [50]. Studies have proved that the intracellular concentrations of antiretrovirals were significantly lower in macrophages than in T lymphocytes [51]. In this purview, THP-1 Mo/Mac cells were used to examine the uptake, cytotoxicity and ROS-induced fluorescence of efavirenz when delivered both as pure form and via liposomes with and without GSH.

Firstly, intracellular lysate concentrations revealed a significantly higher amount of liposomal EFA taken up by the cells in comparison to pure form. The choice of co-delivering GSH was made on the basis of the evidence that supplementing GSH will result in a restoration of intracellular rGSH and a corresponding restoration of macrophage functions [2,6,52]. Earlier studies also suggest that GSH-liposomes were the most protective since they increased cell viability from 28% (blank liposomes) to 67% when encapsulated within liposomes [53]. Thus, as a supplement to ARV therapy, GSH-replenishing agents may attenuate the toxic effects of HAART. Previously, it has been demonstrated that the administration of high doses of GSH was able to reduce the progression of the disease, providing additional effects to azidothymidine (AZT) therapy using a murine model [54,55]. As depicted in Figures 1 and 2, the presence of GSH (depicted as EFA + GSH) enhanced the uptake of EFA when the cells were treated either in pure form (2.46 fold) or in liposomal form (1.68 fold). Liposome delivery to such pathogen reservoirs has received successful attention in the past few years, which is also thoroughly reviewed and discussed [19,26].

In our study, EFA liposomes were negatively charged with particle size less than 150 nm, which could have led to a higher uptake than the pure form. While greater uptake of small liposomes (<100 nm) by mononuclear phagocytic system MPS cells has been reported in the literature [56], various other studies have shown liposome uptake by MPS cells to be improved with increased size [19].

Second, MTS assay data further revealed that encapsulation of EFA into liposomes could be an alternative approach to conventional EFA forms to reduce cytotoxicity; results demonstrated 15.2 fold increase in the viability, thereby improving the proliferation of non-infected THP-1 cells. This increase in the cell viability appears to be due to encapsulation into lipid vesicles based on the assumption that liposome membranes either fuse with cell membranes or are endocytosed by the cells and release their content intracellularly [57]. This mechanism could also depend on the particular liposome formulation, thereby suggesting that negative charge improved endocytosis. Additionally, the antioxidant defense system, due to the presence of GSH within the liposomal vesicle, could have led to a reduction of oxidative stress (Figure 3). Supporting the results of quantitative uptake and MTS assay, confocal images displayed an increase in proliferation of cells as seen with R123 labeled liposomes (Figure 5A,B), thus confirming the elimination of cytotoxicity of EFA at a concentration of 400 μM and a further improvement in the presence of GSH (100 μM).

Finally, flow cytometry data reveal that fluorescence intensity was higher when the cells were treated with pure EFA. Loading of EFA into liposomes was effective in reducing the intracellular accumulation of ROS with a synergistic effect by additional GSH (2.5 fold). This finding suggests that the presence of glutathione moieties within liposomes could lead to an increase in the cellular uptake of liposomes. The results of measurement of the reduction in oxidative stress demonstrated that the anti-oxidative effect of GSH was not impaired by the liposome carrier system and its encapsulation along with EFA within the lipid layers. Furthermore, EFA loaded into liposomes along with GSH exhibited generally a small but significant increased activity in comparison with the same concentration of free forms. Thus encapsulation of glutathione and efavirenz in liposomes apparently facilitate intracellular delivery and prolong the retention time of entrapped agents inside the cell. Nonetheless, co-administration of GSH favoured the reduction of ROS fluorescence when delivered via liposomes form to a greater extent than in the pure form of EFA + GSH (Figures 4 and 5).

Studies using three different humanized mouse models demonstrated provided important insights confirming the roles of macrophages, and other myeloid cells can sustain HIV replication and produce HIV in vivo in the absence of CD4 T cells [58,59]. These study findings indicate that macrophages can serve as a genuine target for HIV infection in vivo by sustaining and transmitting HIV infection [59].

Honeycutt et al. also determined the specific roles of macrophages in HIV persistence during long-term suppressive ART [60]. Their study findings reveal that ART induced the suppression of HIV infection in tissue macrophages, as evident from a significant decrease in the plasma viral load and a reduction in the levels of cell-associated virus. There was no viral rebound in the plasma of the majority of the ART-treated animals’ several weeks after ART interruption. However, in a small subset of animals, a delayed viral rebound was observed that is consistent with the establishment of persistent infection in tissue macrophages [60]. These observations further signify the persistence of HIV in tissue macrophages in vivo.

The data presented in this manuscript are findings from our initial studies exclusively using differentiated human macrophage cell lines. While the uptake of formulations, toxicity, oxidative stress, and GSH levels in macrophages were thoroughly investigated, our compelling results have propelled us to continue future efficacy studies in HIV-infected cells such as monocytes, dendritic cells and CD4 T cells. Both in vitro studies using isolated CD4 T cells, dendritic cells and monocytes from the peripheral blood and in vivo studies using humanized mice will be performed to further characterize the effects of these formulations against HIV.

## 5. Conclusions

In summary, our study provided significant data as follows (i) treatment with EFA and GSH in combination could enhance the uptake and reduce cytotoxicity (ii) encapsulation of EFA into liposomes increased its levels in the macrophages further enhanced in the presence of GSH; (iii) co-delivery of EFA + GSH quenched the intracellular ROS, significantly higher when delivered via liposomes. Combination of EFA and GSH contains advantages; hence, supplementation with GSH recommends being a safe and cost-effective treatment to slow the development of HIV infection and produce an immune-enhancing effect. Although a robust understanding of GSH role in combination with EFA has to be studied using HIV-infected macrophages, our primary attempt using normal macrophages clearly demonstrated the beneficial effect of GSH. Maintaining flexibility and focus on the therapeutic goals for this highly dynamic antiretroviral treatment process is the key to victory in improving the outcome of HIV disease worldwide. To date, there is no product for an antiretroviral drug in combination with either antioxidants or immune modulators accepted as yet. Hence this study could add to the database to evaluate the beneficial use of GSH as an immunomodulator in the treatment of HIV/AIDS. However, the future goal is to evaluate the combination of EFA + GSH in HIV infected THP-1 Mo/Mac cells.

## Figures and Tables

**Table 1.** Composition formula of liposomes loaded with efavirenz.

Formula Code	Lipid	Drug: Lipid: Sterol: T-80 (*w/v*)	Physical Appearance (Post 24 h)
E1	SPC	1: 2:0.5:0.1	Coagulation
E2	DMPC	1: 2:0.5:0.1	Coagulation
E3	DSPC	1: 2:0.5:0.1	Coagulation
F	DMPC/DMPG	1:5/15: 0.1:0	Milky suspension (stable)

**Table 2.** Formulation and characterization data of efavirenz liposomes loaded with and without glutathione.

Formula Code	EFA: GSH (mM)	Size (nm)	Zeta Potential (mV)	Encapsulation Efficiency (%)	
				EFA	GSH
F1	3.17: 0.0	127.7 ± 12.98	−93.8 ± 3.56	95.63 ± 3.91	-
F2	3.17: 0.95	205.03 ± 11.59	−89.2 ± 2.58	96.23 ± 3.45	43.5 ± 4.87

## References

[1] World Health Organization. 2020 04232020. Available online: https://www.who.int/hiv/data/en/ (accessed on 15 September 2021).

[2] Morris D; Guerra C; Khurasany M; Guilford F; Saviola B; Huang Y; Venketaraman V Glutathione supplementation improves macrophage functions in HIV. J. Interferon Cytokine Res 2013, 33, 270–279. 23409922 10.1089/jir.2012.0103

[3] Douek DC; Brenchley JM; Betts MR; Ambrozak DR; Hill BJ; Okamoto Y; Casazza JP; Kuruppu J; Kunstman K; Wolinsky S; HIV preferentially infects HIV-specific CD4+ T cells. Nature 2002, 417, 95–98. 11986671 10.1038/417095a

[4] Yew WW; Chan DP; Chang KC; Zhang Y Does oxidative stress contribute to antituberculosis drug resistance? J. Thorac. Dis 2019, 11, E100–E102. 31463157 10.21037/jtd.2019.06.36 PMC6688019

[5] Beck MA; Handy J; Levander OA The role of oxidative stress in viral infections. Ann. N. Y. Acad. Sci 2000, 917, 906–912. 11268420 10.1111/j.1749-6632.2000.tb05456.x

[6] Venketaraman V; Dayaram YK; Amin AG; Ngo R; Green RM; Talaue MT; Mann J; Connell ND Role of glutathione in macrophage control of mycobacteria. Infect. Immun 2003, 71, 1864–1871. 12654802 10.1128/IAI.71.4.1864-1871.2003 PMC152031

[7] Venketaraman V; Rodgers T; Linares R; Reilly N; Swaminathan S; Hom D; Millman AC; Wallis R; Connell ND Glutathione and growth inhibition of Mycobacterium tuberculosis in healthy and HIV infected subjects. AIDS Res. Ther 2006, 3, 5. 16504020 10.1186/1742-6405-3-5 PMC1397854

[8] World Health Organization. Scaling up Antiretroviral Therapy in Resource-Limited Settings: Treatment Guidelines for a Public Health Approach; Accessed August 2; World Health Organization: Geneva, Switzerland, 2004.

[9] Apostolova N; Blas-Garcia A; Galindo MJ; Esplugues JV Efavirenz: What is known about the cellular mechanisms responsible for its adverse effects. Eur. J. Pharmacol 2017, 812, 163–173. 28690189 10.1016/j.ejphar.2017.07.016

[10] Guerra C; Morris D; Sipin A; Kung S; Franklin M; Gray D; Tanzil M; Guilford F; Khasawneh FT; Venketaraman V Glutathione and adaptive immune responses against Mycobacterium tuberculosis infection in healthy and HIV infected individuals. PLoS ONE 2011, 6, e28378. 22164280 10.1371/journal.pone.0028378 PMC3229597

[11] Morris D; Khurasany M; Nguyen T; Kim J; Guilford F; Mehta R; Gray D; Saviola B; Venketaraman V Glutathione and infection. Biochim. Biophys Acta 2013, 1830, 3329–3349. 23089304 10.1016/j.bbagen.2012.10.012

[12] Wendel A; Cikryt P The level and half-life of glutathione in human plasma. FEBS Lett 1980, 120, 209–211. 7439398 10.1016/0014-5793(80)80299-7

[13] Lagman M; Ly J; Saing T; Kaur Singh M; Vera Tudela E; Morris D; Chi PT; Ochoa C; Sathananthan A; Venketaraman V Investigating the causes for decreased levels of glutathione in individuals with type II diabetes. PLoS ONE 2015, 10, e0118436. 25790445 10.1371/journal.pone.0118436 PMC4366217

[14] Ly J; Lagman M; Saing T; Singh MK; Tudela EV; Morris D; Anderson J; Daliva J; Ochoa C; Patel N; Liposomal Glutathione Supplementation Restores TH1 Cytokine Response to Mycobacterium tuberculosis Infection in HIV-Infected Individuals. J. Interferon Cytokine Res 2015, 35, 875–887. 26133750 10.1089/jir.2014.0210 PMC4642835

[15] Fraternale A; Paoletti MF; Dominici S; Caputo A; Castaldello A; Millo E; Brocca-Cofano E; Smietana M; Clayette P; Oiry J; The increase in intra-macrophage thiols induced by new pro-GSH molecules directs the Th1 skewing in ovalbumin immunized mice. Vaccine 2010, 28, 7676–7682. 20875491 10.1016/j.vaccine.2010.09.033

[16] Fraternale A; Paoletti MF; Casabianca A; Orlandi C; Schiavano GF; Chiarantini L; Clayette P; Oiry J; Vogel JU; Cinatl J Jr.; Inhibition of murine AIDS by pro-glutathione (GSH) molecules. Antivir. Res 2008, 77, 120–127. 18164447 10.1016/j.antiviral.2007.11.004

[17] Fraternale A; Paoletti MF; Casabianca A; Oiry J; Clayette P; Vogel JU; Cinatl J Jr.; Palamara AT; Sgarbanti R; Garaci E; Antiviral and immunomodulatory properties of new pro-glutathione (GSH) molecules. Curr. Med. Chem 2006, 13, 1749–1755. 16787218 10.2174/092986706777452542

[18] Felts RL; Narayan K; Estes JD; Shi D; Trubey CM; Fu J; Hartnell LM; Ruthel GT; Schneider DK; Nagashima K; 3D visualization of HIV transfer at the virological synapse between dendritic cells and T cells. Proc. Natl. Acad. Sci. USA 2010, 107, 13336–13341. 20624966 10.1073/pnas.1003040107 PMC2922156

[19] Kelly C; Jefferies C; Cryan S-A Targeted liposomal drug delivery to monocytes and macrophages. J. Drug Deliv 2011, 2011, 727241. 21512579 10.1155/2011/727241 PMC3065850

[20] Weissig V; Pettinger TK; Murdock N Nanopharmaceuticals (part 1): Products on the market. Int. J. Nanomed 2014, 9, 4357–4373. 10.2147/IJN.S46900 PMC4172146 25258527

[21] Fernandes E; Soares TB; Gonçalves H; Lúcio M Spectroscopic Studies as a Toolbox for Biophysical and Chemical Characterization of Lipid-Based Nanotherapeutics. Front. Chem 2018, 6, 323. 30109226 10.3389/fchem.2018.00323 PMC6080416

[22] Faria MJ; Lopes CM; das Neves J; Lúcio M Lipid Nanocarriers for Anti-HIV Therapeutics: A Focus on Physicochemical Properties and Biotechnological Advances. Pharmaceutics 2021, 13, 1294. 34452255 10.3390/pharmaceutics13081294 PMC8398060

[23] Qi J; Zhuang J; Lu Y; Dong X; Zhao W; Wu W In vivo fate of lipid-based nanoparticles. Drug Discov. Today 2017, 22, 166–172. 27713035 10.1016/j.drudis.2016.09.024

[24] Katragadda ABR; Betageri G Effect of liposome composition and cholesterol on the cellular uptake of stavudine by human monocyte/macrophages. Cell Mol. Biol. Lett 2000, 5, 483–494.

[25] Levy JA; Shimabukuro J; McHugh T; Casavant C; Stites D; Oshiro L AIDS-associated retroviruses (ARV) can productively infect other cells besides human T helper cells. Virology 1985, 147, 441–448. 2416120 10.1016/0042-6822(85)90146-1

[26] Chopra S; Venkatesan N; Betageri GV Liposomes as nanocarriers for anti-HIV therapy. Drug Deliv. Transl. Res 2013, 3, 471–478. 25788354 10.1007/s13346-013-0134-2

[27] Zhao YX; Guo CL; Yao WT; Cai QQ; Wang YS; Wang JQ Vitamin E TPGS based liposomal delivery of doxorubicin in osteosarcoma cancer cells. Biomed. Res 2017, 28, 1344–1349.

[28] Vanaja K; Rani RHS; Sacchidananda S Formulation and Clinical Evaluation Of Ultradeformable Liposomes in the Topical Treatment of Psoriasis. Clin. Res. Regul. Aff 2008, 25, 41–52.

[29] Si F; Shin SH; Biedermann A; Ross GM Estimation of PC12 cell numbers with acid phosphatase assay and mitochondrial dehydrogenase assay: Dopamine interferes with assay based on tetrazolium. Exp. Brain Res 1999, 124, 145–150. 9928836 10.1007/s002210050608

[30] Alberts B; Johnson A; Lewis J; Raff M; Roberts K; Walter P Molecular Biology of the Cell, 4th ed.; The Lipid Bilayer; Garland Science: New York, NY, USA, 2002.

[31] Makabi-Panzu B; Gourde P; Désormeaux A; Bergeron MG Intracellular and serum stability of liposomal 2’,3’-dideoxycytidine. Effect of lipid composition. Cell Mol. Biol 1998, 44, 277–284. 9593578

[32] Wang Y; de Clercq E; Li G Current and emerging non-nucleoside reverse transcriptase inhibitors (NNRTIs) for HIV-1 treatment. Expert Opin. Drug Metab. Toxicol 2019, 15, 813–829. 31556749 10.1080/17425255.2019.1673367

[33] Jin J; Grimmig B; Izzo J; Brown LAM; Hudson C; Smith AJ; Tan J; Bickford PC; Giunta B HIV Non-Nucleoside Reverse Transcriptase Inhibitor Efavirenz Reduces Neural Stem Cell Proliferation In Vitro and In Vivo. Cell Transplant 2016, 25, 1967–1977. 28836850 10.3727/096368916X691457 PMC5683847

[34] Ivanov AV; Valuev-Elliston VT; Ivanova ON; Kochetkov SN; Starodubova ES; Bartosch B; Isaguliants MG Oxidative Stress during HIV Infection: Mechanisms and Consequences. Oxid. Med. Cell Longev 2016, 2016, 8910396. 27829986 10.1155/2016/8910396 PMC5088339

[35] Bendich A 15—Role of Antioxidants in the Maintenance of Immune Functions. In Natural Antioxidants in Human Health and Disease; Frei B, Ed.; Academic Press: San Diego, CA, USA, 1994; pp. 447–467.

[36] Singh G; Pai RS Dawn of antioxidants and immune modulators to stop HIV-progression and boost the immune system in HIV/AIDS patients: An updated comprehensive and critical review. Pharmacol. Rep 2015, 67, 600–605. 25933975 10.1016/j.pharep.2014.12.007

[37] Poli G; Bressler P; Kinter A; Duh E; Timmer WC; Rabson A; Justement JS; Stanley S; Fauci AS Interleukin 6 induces human immunodeficiency virus expression in infected monocytic cells alone and in synergy with tumor necrosis factor alpha by transcriptional and post-transcriptional mechanisms. J. Exp. Med 1990, 172, 151–158. 2193094 10.1084/jem.172.1.151 PMC2188185

[38] Venketaraman V; Kaushal D; Saviola B Mycobacterium tuberculosis. J. Immunol. Res 2015, 1–2. 10.1155/2015/857598 PMC4539972 26345333

[39] Anddre Valdivia JL; Gonzalez L; Hussain P; Saing T; Islamoglu H; Pearce D; Ochoa C; Venketaraman V Restoring Cytokine Balance in HIV-Positive Individuals with Low CD4 T Cell Counts. AIDS Res. Hum. Retrovir 2017, 33, 905–918. 28398068 10.1089/aid.2016.0303 PMC5576219

[40] Muller CE Prodrug approaches for enhancing the bioavailability of drugs with low solubility. Chem. Biodivers 2009, 6, 2071–2083. 19937841 10.1002/cbdv.200900114

[41] Makoni PA; Kasongo KW; Walker RB Short Term Stability Testing of Efavirenz-Loaded Solid Lipid Nanoparticle (SLN) and Nanostructured Lipid Carrier (NLC) Dispersions. Pharmaceutics 2019, 11, 397. 10.3390/pharmaceutics11080397 PMC6723231 31398820

[42] Varshosaz J; Taymouri S; Jahanian-Najafabadi A; Alizadeh A Efavirenz oral delivery via lipid nanocapsules: Formulation, optimisation, and ex-vivo gut permeation study. IET Nanobiotechnol 2018, 12, 795–806. 30104454 10.1049/iet-nbt.2018.0006 PMC8675992

[43] Kamboj S; Sethi S; Rana V Lipid based delivery of Efavirenz: An answer to its erratic absorption and food effect. Eur. J. Pharm. Sci 2018, 123, 199–216. 30031861 10.1016/j.ejps.2018.07.037

[44] Pokharkar V; Patil-Gadhe A; Palla P Efavirenz loaded nanostructured lipid carrier engineered for brain targeting through intranasal route: In-vivo pharmacokinetic and toxicity study. Biomed. Pharm 2017, 94, 150–164. 10.1016/j.biopha.2017.07.067 28759752

[45] Senapati PC; Sahoo SK; Sahu AN Mixed surfactant based (SNEDDS) self-nanoemulsifying drug delivery system presenting efavirenz for enhancement of oral bioavailability. Biomed. Pharm 2016, 80, 42–51. 10.1016/j.biopha.2016.02.039 27133038

[46] Ramana LN; Sethuraman S; Ranga U; Krishnan UM Development of a liposomal nanodelivery system for nevirapine. J. Biomed. Sci 2010, 17, 57. 20624325 10.1186/1423-0127-17-57 PMC2914021

[47] Cagdas FM; Ertugral N; Bucak S; Atay NZ Effect of preparation method and cholesterol on drug encapsulation studies by phospholipid liposomes. Pharm. Dev. Technol 2011, 16, 408–414. 20433249 10.3109/10837451003774401

[48] Vanaja K; Wahl MA; Bukarica L; Heinle H Liposomes as carriers of the lipid soluble antioxidant resveratrol: Evaluation of amelioration of oxidative stress by additional antioxidant vitamin. Life Sci 2013, 93, 917–923. 24177602 10.1016/j.lfs.2013.10.019

[49] Carter CA; Ehrlich LS Cell biology of HIV-1 infection of macrophages. Annu. Rev. Microbiol 2008, 62, 425–443. 18785842 10.1146/annurev.micro.62.081307.162758

[50] Nasr N; Maddocks S; Turville SG; Harman AN; Woolger N; Helbig KJ; Wilkinson J; Bye CR; Wright TK; Rambukwelle D; HIV-1 infection of human macrophages directly induces viperin which inhibits viral production. Blood 2012, 120, 778–788. 22677126 10.1182/blood-2012-01-407395

[51] Jin X; McGrath MS; Xu H Inhibition of HIV Expression and Integration in Macrophages by Methylglyoxal-BisGuanylhydrazone. J. Virol 2015, 89, 11176–11189. 26223636 10.1128/JVI.01692-15 PMC4645666

[52] Teskey G; Abrahem R; Cao R; Gyurjian K; Islamoglu H; Lucero M; Martinez A; Paredes E; Salaiz O; Robinson B; Glutathione as a Marker for Human Disease. Adv. Clin. Chem 2018, 87, 141–159. 30342710 10.1016/bs.acc.2018.07.004

[53] Paromov V; Kumari S; Brannon M; Kanaparthy NS; Yang H; Smith MG; Stone WL Protective Effect of Liposome-Encapsulated Glutathione in a Human Epidermal Model Exposed to a Mustard Gas Analog. J. Toxicol 2011, 2011, 109516. 21776256 10.1155/2011/109516 PMC3135079

[54] Magnani M; Fraternale A; Casabianca A; Schiavano GF; Chiarantini L; Palamara AT; Ciriolo MR; Rotilio G; Garaci E Antiretroviral effect of combined zidovudine and reduced glutathione therapy in murine AIDS. AIDS Res. Hum. Retrovir 1997, 13, 1093–1099. 9282814 10.1089/aid.1997.13.1093

[55] Fraternale A; Casabianca A; Tonelli A; Chiarantini L; Brandi G; Magnani M New drug combinations for the treatment of murine AIDS and macrophage protection. Eur. J. Clin. Investig 2001, 31, 248–252. 11264653 10.1046/j.1365-2362.2001.00806.x

[56] Ahsan F; Rivas IP; Khan MA; Torres Suarez AI Targeting to macrophages: Role of physicochemical properties of particulate carriers–liposomes and microspheres–on the phagocytosis by macrophages. J. Control Release 2002, 79, 29–40. 11853916 10.1016/s0168-3659(01)00549-1

[57] Caddeo C; Teskac K; Sinico C; Kristl J Effect of resveratrol incorporated in liposomes on proliferation and UV-B protection of cells. Int. J. Pharm 2008, 363, 183–191. 18718515 10.1016/j.ijpharm.2008.07.024

[58] Honeycutt JB; Liao B; Nixon CC; Cleary RA; Thayer WO; Birath SL; Swanson MD; Sheridan P; Zakharova O; Prince F; T cells establish and maintain CNS viral infection in HIV-infected humanized mice. J. Clin. Investig 2018, 128, 2862–2876. 29863499 10.1172/JCI98968 PMC6026008

[59] Honeycutt JB; Wahl A; Baker C; Spagnuolo RA; Foster J; Zakharova O; Wietgrefe S; Caro-Vegas C; Madden V; Sharpe G; Macrophages sustain HIV replication in vivo independently of T cells. J. Clin. Investig 2016, 126, 1353–1366. 26950420 10.1172/JCI84456 PMC4811134

[60] Honeycutt JB; Thayer WO; Baker CE; Ribeiro RM; Lada SM; Cao Y; Cleary RA; Hudgens MG; Richman DD; Garcia JV HIV persistence in tissue macrophages of humanized myeloid-only mice during antiretroviral therapy. Nat. Med 2017, 23, 638–643. 28414330 10.1038/nm.4319 PMC5419854

